# RNA-extraction-free diagnostic method to detect SARS-CoV-2: an assessment from two states, India

**DOI:** 10.1017/S0950268821002302

**Published:** 2021-11-02

**Authors:** Madhumathi Jayaprakasam, Sumit Aggarwal, Arati Mane, Vandana Saxena, Amrita Rao, Bhaswati Bandopadhyay, Banya Chakraborty, Subhasish Kamal Guha, Mekhala Taraphdar, Alisha Acharya, Bishal Gupta, Sonia Deb, Aparna Chowdhury, Kh Jitenkumar Singh, Prashant Tapase, Ravindra M. Pandey, Balram Bhargava, Samiran Panda

**Affiliations:** 1 Division of Epidemiology and Communicable Diseases, Indian Council of Medical research (ICMR), Ansari Nagar, New Delhi, India; 2 ICMR-National AIDS Research Institute (NARI), Pune, India; 3 Department of Microbiology, School of Tropical Medicine (STM), Kolkata, India; 4 ICMR-National Institute of Medical Statistics (NIMS), Ansari Nagar, India; 5 Department of Biostatistics, All India Institute of Medical Sciences (AIIMS), New Delhi, India; 6 School of Tropical Medicine (STM), Kolkata, India; 7 Indian Council of Medical Research (ICMR), Ansari Nagar, New Delhi, India

**Keywords:** Diagnostic test, Dry swab, Heat-inactivation, Performance-evaluation, RNA-extraction-free method, SARS-CoV-2

## Abstract

With increasing demand for large numbers of testing during the coronavirus disease 2019 pandemic, alternative protocols were developed with shortened turn-around time. We evaluated the performance of such a protocol wherein 1138 consecutive clinic attendees were enrolled; 584 and 554 respectively from two independent study sites in the cities of Pune and Kolkata. Paired nasopharyngeal and oropharyngeal swabs were tested by using both reference and index methods in a blinded fashion. Prior to conducting real-time polymerase chain reaction, swabs collected in viral transport medium (VTM) were processed for RNA extraction (reference method) and swabs collected in a dry tube without VTM were incubated in Tris–EDTA–proteinase K buffer for 30 min and heat-inactivated at 98 °C for 6 min (index method). Overall sensitivity and specificity of the index method were 78.9% (95% confidence interval (CI) 71–86) and 99% (95% CI 98–99.6), respectively. Agreement between the index and reference method was 96.8% (*k* = 0.83, s.e. = 0.03). The reference method exhibited an enhanced detection of viral genes (E, N and RNA-dependent RNA polymerase) with lower Ct values compared to the index method. The index method can be used for detecting severe acute respiratory syndrome corona virus-2 infection with an appropriately chosen primer–probe set and heat treatment approach in pressing time; low sensitivity constrains its potential wider use.

## Introduction

The coronavirus disease 2019 (COVID-19) pandemic swept through the world with unprecedented speed and impact on lives and livelihoods [1]. Within 4 months of its onset, more than 118 000 cases and 4291 deaths were reported from 114 countries. All of these happened following an outbreak of ‘unusual cases of pneumonia’ notified for the first time from the Wuhan city of Hubei province, China in December 2019 [2]. Such a rapid spread of the causative virus severe acute respiratory syndrome corona virus-2 (SARS-CoV-2) reminded humankind of the influenza pandemic causing havoc about 100 year ago [3, 4]. Developing simple and reliable diagnostic tests appeared paramount in this context as care service-related needs escalated and demand for tools to conduct quick screening and survey also increased [5].

As with many other infectious diseases, SARS-CoV-2 infection is detected reliably by the real-time polymerase chain reaction (RT-PCR) as it is a highly sensitive and specific tool [6]. Although the Center for Disease Control (CDC), USA recommended the gene targets for two nucleocapsid proteins (N1 and N2) of SARS-CoV-2 for diagnostic assays [7], the World Health Organization proposed using envelope (E) gene target for first-line screening and RNA-dependent RNA polymerase (RdRP) for confirmation [8, 9]. Notably, assays using E and N2 gene primers were found to be more sensitive [10]. The combination of two gene targets is recommended to enhance the accuracy of diagnosis in the context of possible viral genetic variability: one from the conserved region of the virus and another from SARS CoV-2 specific region of the genome [8].

Several alternative protocols described ways to simplify the RT-PCR test by excluding the RNA extraction step [11–14]. These modifications attempted to reduce the turn-around time for quickly obtaining test results and also to address the issue of shortage of RNA extraction kits when the demand runs high. Heating of nasopharyngeal swab specimens in transport medium and skipping the RNA extraction step before proceeding to conduct RT-PCR has been reported to be fast and reliable [12]. Direct heating of viral extracts from swab specimens for 5 min at 98 °C resulted in 97% sensitivity and 100% specificity when examined against purified RNA as the gold standard [15]. Direct RT-PCR assay with heat-inactivated or lysed samples using generic buffers such as Tris or Tris-EDTA (TE) served as an effective alternative method [16]. A similar approach to RT-PCR, using heat-inactivated TE buffer extract of nasopharyngeal swabs transported in a dry tube from the sample collection site to the laboratory, has been described from India as well [17]. However, utility of this method and modified version of it as suggested by the Centre for Cellular and Molecular Biology, Hyderabad, India in a real-world programme setting was not examined. This modification was in line with the study of Chu *et al*. [13] for SARS-CoV-2 and de Paula *et al*. [18] for hepatitis A virus where proteinase K was used along with TE buffer. We assessed the performance of the modified version of the test approach of Kiran *et al*., in the programme setting for diagnosis of COVID-19, using E, RdRP and N primer–probe-based assay [17].

## Methods

The current investigation took place during 10th November through 11th December 2020. The proposal for evaluation was developed in early October 2020 and approval was obtained from the Central Ethics Committee for Human Research (CECHR) of Indian Council of Medical Research (ICMR) on 30th October 2020. Written informed consent was obtained from individuals consenting to participate in this study.

### Study settings and participants

The present investigation was conducted at two sites in India namely, the ICMR-National AIDS Research Institute (ICMR-NARI), Pune in the western state of Maharashtra and the School of Tropical Medicine (STM), Kolkata in the eastern state of West Bengal. Necessary approvals were obtained from the Ethics Committees of these two respective institutes as well. Consecutive clinic attendees (⩾18 year of age) at the designated study sites, who came for SARS-CoV-2 testing, were invited to participate in this investigation.

### Implementation

Each consenting clinic attendee was registered on a web-based portal maintained by ICMR with a specimen referral form (SRF) number created at the collection site, which was used for labelling the viral transport medium (VTM) containing tube. In order to ensure blinding, a different set of unique codes was randomly generated from the ICMR-headquarter, New Delhi for each study site using an Excel-based tool for labelling the corresponding swabs collected and placed in dry tubes. The link page, containing ICMR SRF number and the paired unique code for swabs in dry tubes for each enrolled participant, was available only with the designated staff at the respective study sites. Blinding was ensured through barring access of the laboratory staff involved in test procedures and generation of test results to the link page.

### Sample collection and processing

‘Specimen Collection, Packaging and Transport Guidelines for 2019 Novel Coronavirus (2019-nCoV)’ was adhered to during study implementation [19]. Two naso-pharyngeal swabs and two oropharyngeal swabs were collected from each of the enrolled participants in single sitting. The swabs (one naso-pharyngeal and one oro-pharyngeal) saved in labelled VTM tubes and those kept in labelled dry tubes, were transported to the participating laboratories and processed on the same day of sample collection.

### Reference test and index test

**Fig. 1.**
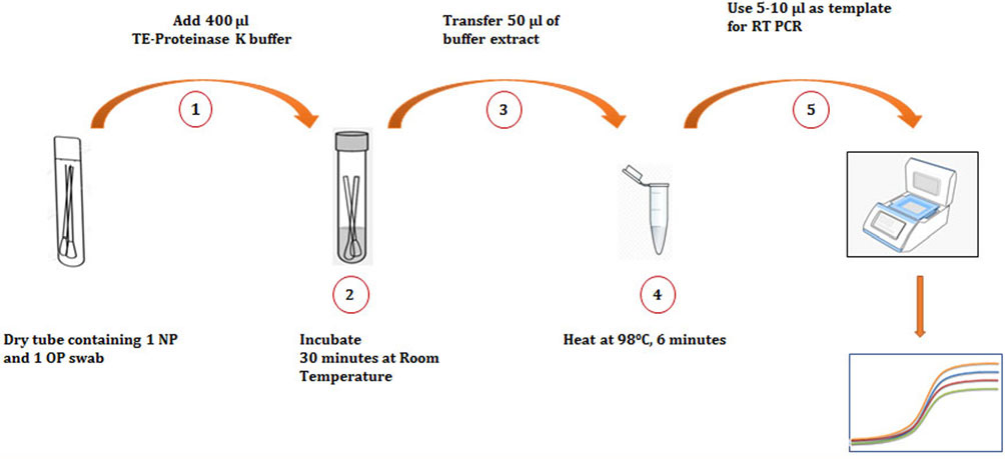
Schematic illustration of a modified dry tube-based heat inactivation method followed by RT-PCR for the detection of SARS-CoV-2. (1) Addition of TE-proteinase K buffer to the tube containing swabs. (2) Incubation of swabs in buffer to extract viral particles. (3) Transfer of viral extract. (4) Inactivation of the virus by heating. (5) Setting up RT-PCR reaction and interpretation of assay. NP, nasopharyngeal swab; OP, oropharyngeal swab.

- *RT-PCR test with swab specimens transported in VTM tube (reference method)*: The reference method used one nasopharyngeal and one oropharyngeal swab (HiMedia™ Laboratories Pvt Ltd, Mumbai, India) collected from each participant and saved in the tube containing 3 ml VTM (HiMedia™ Laboratories Pvt Ltd, Mumbai, India). About 200 μl of the VTM extract was used for RNA extraction followed by RT-PCR assay [9].
- *Dry swab-based RT-PCR (index method)*: One nasopharyngeal swab and one oropharyngeal swab collected from each participant were transported to the laboratories in 10 ml sample collection tubes (HiMedia™ Laboratories Pvt Ltd, Mumbai, India) without adding VTM to them. At the laboratories, 400 μl of Tris–EDTA–proteinase K (TE-P) buffer (10 mm Tris (pH 7.4), 0.1 mm EDTA and 2 mg/ml proteinase K) (Bio Ultra, for molecular biology, Sigma-Aldrich, Bangalore, India) was added to swab specimens transported in dry tubes and incubated for 30 min at room temperature. About 50 μl of the TE-P extract was aliquoted and heat-inactivated at 98 °C for 6 min in a sealed 96 well PCR plate using thermal cycler with a heated lid (ICMR-NARI site) or in 0.5 ml tubes using heat block (STM site). The heat-inactivated extract was then used as a template for RT-PCR reaction (Fig. 1).

### Nucleic acid amplification assay

RNA extraction from clinical specimens transported in VTM tubes was carried out as per instructions accompanying the commercial RNA extraction kit (QIAmp viral RNA Mini Kit, QIAGEN, New Delhi, India). The RT-PCR reaction was carried out using ‘CoviDx mPlex-4R SARS-CoV-2’ (NeoDx Biotech Labs Private Limited, DSS Imagetech, New Delhi, India) as per the manufacturer's instructions with primer–probe sets (Table 1) for the detection of the SARS-CoV-2-specific genes E, N and RdRP. Human RNase P was used as an internal control in this single tube assay. Briefly, a 25 μl reaction was set-up containing an 8 μl template (purified RNA from VTM sample for the reference method and heat-inactivated dry swab lysate for the index method), 12.5 μl of 2× master mix, 1.25 μl 20× primer and probe mix and 3.25 μl nuclease free water. In each assay, a positive control and no template control were included. The RT-PCR assays were conducted on ‘CFX96-IVD Real-time PCR system’ (Bio-Rad Laboratories India Pvt. Ltd., Gurugram, Haryana, India) using the following cycling conditions: 50 °C for 15 min for reverse transcription, 95 °C for 2 min and 45 cycles of 95 °C for 15 s and 58 °C for 30 s.

**Table 1.** Primer probe sets of RT-PCR kit used for assay

Sl. no.	Target	Reporter	Primer sequence	Amplicon size (bp)
1	E gene	FAM	F: ACAGGTACGTTAATAGTTAATAGCGT R: ATATTGCAGCAGTACGCACACA	113
2	RdRp gene	CY5	F: GTGAAATGGTCATGTGTGGCGG R: CAAATGTTAAAAACACTATTAGCATA	100
3	N gene	HEX	F: TTCCCTATGGTGCTAACAAAGACG R: CTTGAGGAAGTTGTAGCACGATTG	129
4	Human Rnase P	TEXAS RED	F: AGATTTGGACCTGCGAGCG R: GAGCGGCTGTCTCCACAAGT	65

### Sample size estimation and data analysis

An earlier evaluation of the RNA-extraction-free dry swab-based RT-PCR method [17] in the clinic setting was conducted by us and estimated to have 56% sensitivity and 95% specificity [20]. The modified index method (described above) was expected to have improved sensitivity and thus we conservatively assumed it to be 75% with minimum acceptance lower confidence limit of 60% based on which the calculated number of cases required was 107 [21]. With the recorded prevalence of 20% SARS-CoV-2 infection among clinic attendees in Pune and Kolkata during the current study, the number of SARS-CoV-2 negative individuals to be included was calculated as 428 [107 × (1 − 0.2/0.2)] = 428; the total estimated sample size being 535.

A cycle threshold (Ct) value of 40 or less was considered as positive. The binary outcome (yes/no), in the form of presence or absence of SARS-CoV-2 infection generated by the index method was assessed against the results obtained following RT-PCR tests on swab specimens transported in VTM. The sensitivity, specificity, concordance, discordance, positive predictive value, negative predictive value and agreement between the tests and their 95% confidence interval (CI) were computed using Stata version 13.1 (StataCorp LP, College Station, TX, USA). GraphPad Prism (version 5) and R-CRAN (version 4.0.3) with the ggplot2 library was used for graphical representations.

## Results

### Participants

Consecutive clinic attendees at the two study sites were enrolled. Although 15 of the 600 (2.5%) attendees at ICMR-NARI, Pune site refused to provide consent, 96 of the 650 attendees at STM, Kolkata (14.8%) did so. Information obtained from 584 participants by ICMR-NARI (one specimen could not be analysed due to inadequate volume) and 554 participants by STM, Kolkata were included in the analyses. Each site thus fulfilled sample size requirement on its own and allowed examination of performance of the index test in two different real-world settings independent of each other and thus fulfilling the criteria of conducting evaluation in different settings.

The majority of the participants were male (767/1138; 67%); age ranging from 18 to 85 year (Table 2). Nearly 30% of the participants were symptomatic (342/1138); most common ones being fever (52%), cough (35%), bodyache (12%), sore throat (7%), breathlessness (3.5%) and anosmia (3.5%).

**Table 2.** Demographic profile of study participants

Characteristics	Frequency (%)
Total number of participants	1138
Male (*n* = 767)	
Mean age ± s.d. (year)	38.47 ± 13.2
Median age (year) [IQR]	37 [27–49]
Female (*n* = 371)	
Mean age ± s.d. (year)	35.97 ± 13.7
Median age (year) [IQR]	34 [24–45]
Age group (years)	Total *n* (%)
18–30	437 (38)
31–40	247 (22)
41–50	235 (21)
51–60	160 (14)
>60	59 (5)
Total	1138 (100)
Symptoms (*n* = 342)	
Cough	120 (35.1)
Fever	178 (52.0)
Bodyache	40 (11.7)
Breathlessness	12 (3.5)
Sore throat	24 (7.0)
Nausea	4 (1.2)
Heamoptysis	1 (0.3)
Vomiting	1 (0.3)
Chest pain	2 (0.6)
Anosmia	12 (3.5)
Others	79 (23.1)

### Comparison of Ct values: reference *vs.* index method

**Fig. 2.**
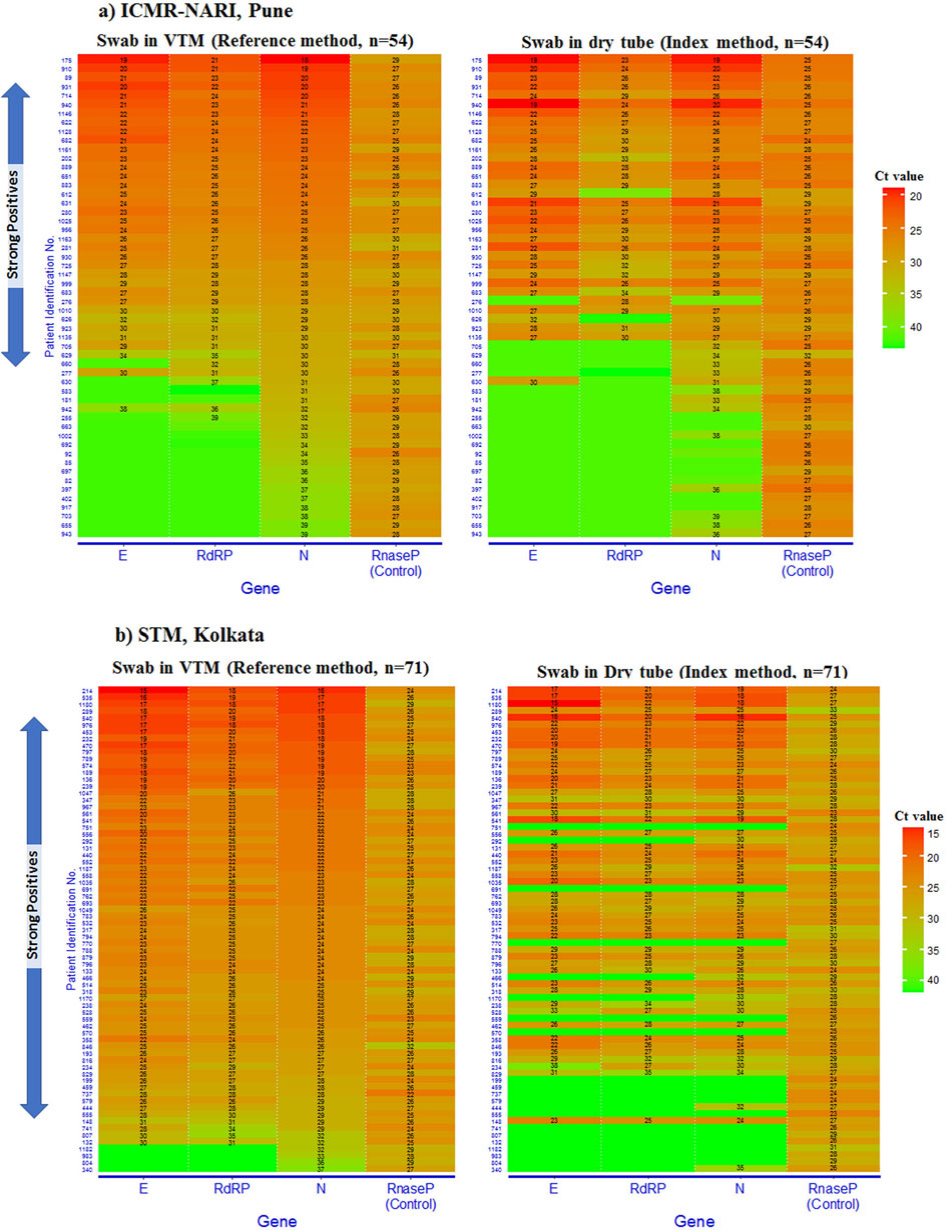
Heat map of cycle threshold (Ct) values for E, RdRP and N genes detected by reference and index methods on clinical samples from ICMR-NARI (*n* = 54) and STM, Kolkata (*n* = 71). The heat map is ranked by N gene Ct. Ct values of human Rnase P used as a control in RT-PCR is shown on the right. A Ct value ⩽40 is considered as positive. Samples that are positive for all three viral genes are indicated as strong positives by an arrow on the left.

Heat-maps of Ct values for E, RdRP and N genes detected by reference and index methods were plotted. Samples, which were detected having at least one of these genes by using the VTM-based method, were used for comparisons and were examined to explore how did the index method performed against them. The N gene primer–probe set showed superior performance compared to the other two genes by both reference and index methods. Figure 2 presents comparative data visualisation with juxtaposition pertaining to the three aforementioned genes along with the internal control (human Rnase P). The reference method could detect either one of the three genes (E, RdRP or N gene) in 54 samples at ICMR-NARI and 71 samples at STM. However, the index method could detect either one of the three genes in 45 out of the aforementioned 54 samples at ICMR-NARI and 55 of the 71 samples at STM (Figs 2a and b). The index method could not detect any of the three target genes in 17% (9/54) of the clinical specimens at ICMR-NARI and the proportion of such missed events had risen to 23% (16/71) at STM. Parity between the reference and extraction-free methods in terms of detecting positive specimens was better at ICMR-NARI (45/54; 83.3%) (Fig. 2a) compared to the results obtained at STM (55/71; 77.5%) (Fig. 2b). This difference could be explained by the difference in heat treatment methods used by the respective centres. Although the STM site used heat block for maintaining 98 °C at 6 min, the ICMR-NARI site had used thermal cycler with heated lid, which comparatively had better yield.

### Distribution of Ct values

**Fig. 3.**
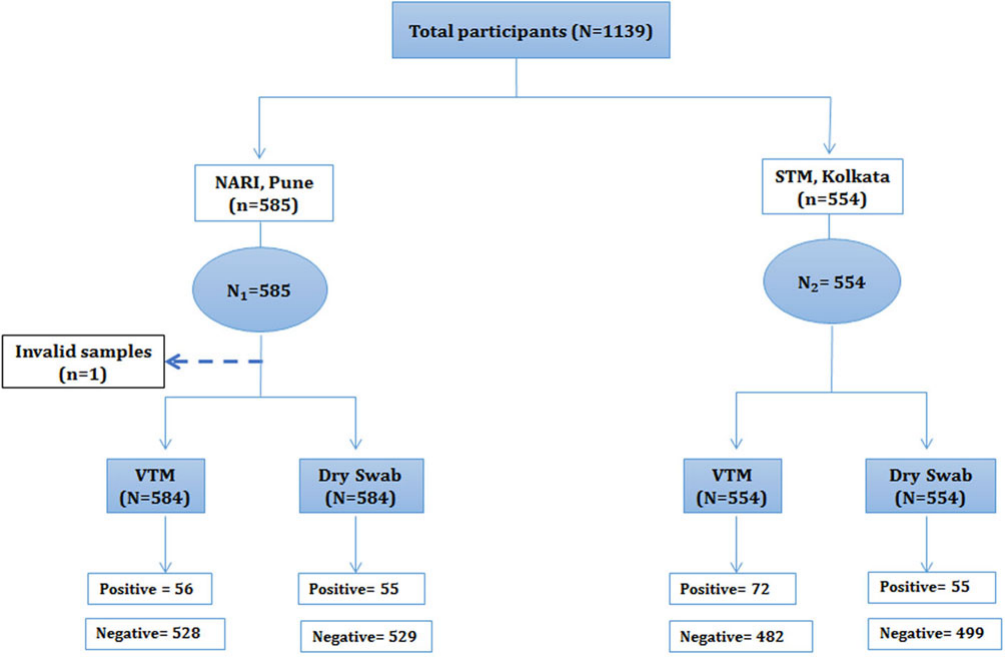
Flowchart showing enrolment of participants at two study sites (ICMR-NARI, Pune, Maharashtra and STM, Kolkata, West Bengal).

We compared matched Ct values generated by using both reference as well as index methods. Samples which were tested positive by the reference method for each gene were used for the analysis of Ct values. Reference method-based RT-PCR results had 1–10 Ct values lower than those generated by the index method for E, RdRP and N genes in more than two-thirds of the samples (Fig. 3). The mean Ct values (±s.d.) for target genes detected by the reference method were as follows: E = 23.69 ± 4.03, RdRP = 25.59 ± 4.06 and N = 25.76 ± 5.33. These values were significantly lower (*P* < 0.0001, Wilcoxon signed rank test), compared to the values generated by the dry swab method (E = 24.42 ± 4.01, RdRP = 26.80 ± 3.32 and N = 26.61 ± 4.87).

### Performance of index method

**Fig. 4.**
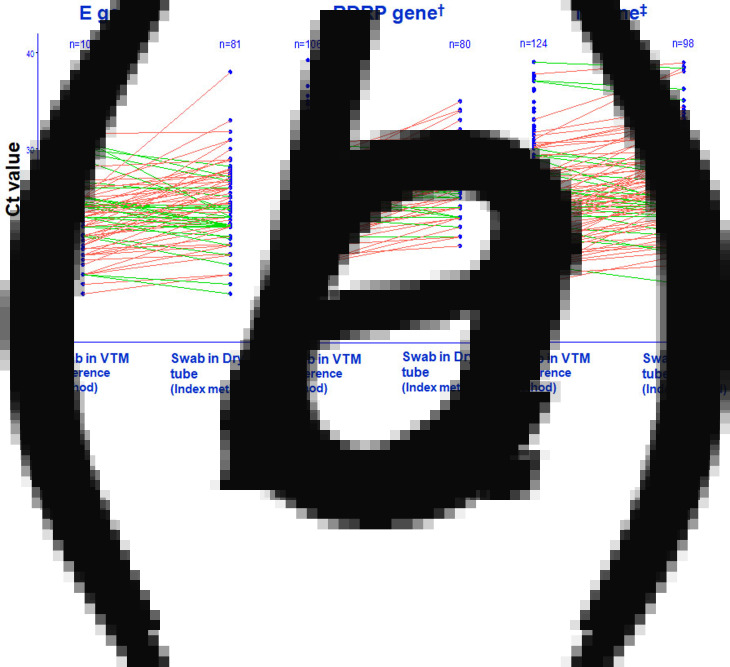
Scatter plot of Ct values for matched samples tested by reference and index methods for (a) E gene (b) RdRP gene and (c) N gene. *Of 103 samples having E gene detection through VTM, 81 were detected through the index method. ^†^Of 106 samples having RdRP gene detection through VTM, 80 were detected through the index method. ^‡^Of 124 samples having N gene detection through VTM, 98 were detected through the index method.

Although 11% (128/1138) of the total clinic attendees tested positive for SARS-CoV-2 infection by the reference method, the index method involving the transportation of swabs in a dry tube environment detected 78% (101/128) of them thus reducing the overall detection to 8.8% (101/1138). Of the 584 samples tested at the ICMR-NARI site, overall 9.6% samples (56/584) tested positive by the reference method and 9.4% (55/584) were positive by the index method. Of the 554 samples tested at the STM, Kolkata, 72 (13%) were detected as positive by the reference method and 55 (10%) by the index method (Fig. 4). The overall sensitivity of the index method was 78.9% (95% CI 71–86) and specificity was 99% (95% CI 98–99.6). The observed overall agreement between the index and reference methods was 96.8% and the discordance was 3.16%; kappa value (*κ*) was 0.83 (95% CI 0.77–0.89, s.e. = 0.030). Site disaggregated data are presented in Table 3.

**Table 3.** Performance of the RNA extraction-free index method in current investigation

	Study site		Overall
	NARI, Pune	STM, Kolkata	
Sensitivity (%) (95% CI)	82 (70–91)	76.4 (65–86)	78.90 (71–86)
Specificity (%) (95% CI)	98.3 (97–99)	100 (99–100)	99 (98–99.6)
Positive predictive value (%) (95% CI)	83.6 (71–92)	100 (94–100)	92 (85–96)
Negative predictive value (%) (95% CI)	98 (97–99)	96.6 (95–98)	97 (96–98)

## Discussion

Conducting research during outbreak situation faces many challenges. Lengthy start-up period before one could carry out observational research in pandemic situation has been cited as one of these challenges [22], and the other challenges are reactive approaches, socio-political pressures to approve repurposed or promising drugs [23] or diagnostic kits and urgency of the researchers to inform public health decisions. Besides prompt implementation against such background, the strength of the current investigation rests with its methodology. First, a study population akin to the individuals, on which the index method could be applied in future, was assembled. Second, both the reference as well as index methods pertaining to SARS-CoV-2 diagnosis were applied to all the study participants and laboratory investigators remained blinded to such assignments at both the study sites, which independently conducted their investigations.

With increasing demand for testing in pandemic situation, several researchers have explored the possibility of utilising alternative specimen collection procedures, processing steps and testing methods. Direct heating of nasopharyngeal swab specimens in universal viral transport medium at 65 °C for 10 min without RNA extraction reportedly yielded sensitivity comparable to the standard method [14]. On the contrary, an earlier evaluation of a direct extraction method using buffer eluates of the swabs transported in dry tube (without transport medium) and heat treatment at 98 °C for 6 min against the reference method on 978 clinical samples yielded an overall sensitivity of 56% (95% CI 49.8–61.6) and specificity of 95% (95% CI 93.4–96.8) [20]. Pretreatment of such buffer eluates with proteinase K followed by heat inactivation was found to improve sensitivity in a pilot laboratory assay of SARS-CoV-2 [24], consistent with the previous reports from other researchers [13].

We conducted the current assessment to evaluate a similar approach of direct extraction from dry swabs using TE buffer and proteinase K followed by heat inactivation from nasopharyngeal specimens collected from consecutive clinic attendees. This modification over an earlier version of the test approach [17] increased the overall sensitivity from 56% to 79%.

Contrastingly, Srivatsan *et al*. [25] reported much higher sensitivity (100%) and specificity (99.4%) with direct extraction from dry swabs using low-TE buffer elution, proteinase K pre-treatment and heat inactivation. Notably, the study by Srivatsan *et al*. used archived samples as well as anterior nasal dry swabs collected as convenience specimens. Such designs are prone to introduction of biases that we could avoid by enrolling consecutive clinic attendees from two different clinic settings.

Heat map-based visualisation, in the present investigation, demonstrated that the standard RNA extraction method exhibited an enhanced detection of gene targets with lower Ct values (corollary of higher RNA concentration or viral load in a given condition) compared to the dry swab elution where RNA-fragmentation during heat inactivation remains a possibility, which could lead to reduced sensitivity. The difference could further be explained by purification that takes place during RNA extraction. Moreover, concentration of RNA that is achieved and removal of PCR inhibitory substances during RNA extraction also could contribute to better yield due to intact high-quality RNA available for RT-PCR. Notably, Chen *et al*. reported that there was a 50–66% drop in RNA copy number after heating at 80 °C for 20 min [26] while different inactivation methods were compared. In the current study, the index method failed to detect 17% and 23% of positive specimens at the ICMR-NARI site and STM, Kolkata site, respectively. Notably, eight positive specimens with low Ct values detected by the reference method were missed out by the extraction-free method at STM, Kolkata. This could be due to the compromised quality of RNA during heat inactivation process which failed to amplify all three viral genes. The low detection at STM was not related to site-specific performance issue as the internal control was detected at low Ct values in these specimens. Rather, different heating methods used at two study sites could explain the difference in recorded sensitivity due to resulting difference in time of exposure of samples to 98 °C. Hasan *et al*. compared standard method with direct extraction method and showed an enhanced detection of human RNase P compared to viral genes with a difference of 1–6 Ct values [14]. Burton *et al*. thus recommend local validation [27] of heat-inactivation and examination of its effects on molecular testing as recovery of amplifiable RNA can vary widely over relatively small changes in temperature and time. These observations underline the importance of paying attention to the heat treatment method while using the extraction-free technique for viral diagnostics. In order to ensure consistent performance, it is recommended to utilise thermal cyclers for heat treatment or use a thermocouple for each heating block. Thermal cyclers with heated lid prevent condensation of liquid into the sample, controls sample temperatures precisely and enables uniform temperatures across the thermal block.

Importantly, Smyrlaki *et al*. carried out extensive standardisations of different heat inactivation protocols. The authors reported that all high temperature (⩾95 °C) conditions resulted in similar Ct values and recommended inactivation at 95–98 °C [16]. Complete inactivation of SARS-CoV-2 was observed only after heat treatment at 95 °C for 1 or 5 min in another study [27]. On the contrary, Mallmann *et al*. [24] tested different conditions and reported that pretreatment with proteinase K and heat treatment at 98 °C yielded best results with Ct values similar to that in a standard method; conditions similar to those followed in the current investigation.

It was observed in the current investigation that the detection of SARS-CoV-2 N gene target was superior compared to E and RdRP genes. This is in agreement with the previous reports [28], where N gene-based RT-PCR was shown to be more detectable due to relative abundance of N gene subgenomic mRNA [29]. The primer–probe set for N1 gene showed better performance due to shorter amplicon size in another heat inactivation protocol as well [16]. Hence, we maintain that the primer and probe sets should be carefully chosen if heat inactivation methods are to be deployed. Our study has further highlighted the importance of deploying appropriate heat treatment method if RNA-extraction-free detection technique is to be followed.

In conclusion, the evaluated index method has the potential to serve as an alternative protocol for identifying SARS-CoV-2 infection in resource-limited settings. However, the following observations appear demanding if using this method in programme setting is to be considered: (a) requirement of carefully selected primer and probe sets for better outcome and (b) the necessity of adherence to appropriate heat treatment method as small variation in heating can have a significant impact on test performance. The lower sensitivity of this RNA-extraction-free RT-PCR method in real-world setting appears to be one of its limitations.

## Data Availability

The data presented in this study are available on request from the corresponding author. The data are not publicly available due to ethical reasons.
